# The Antecedents and Consequences of Workaholism: Findings From the Modern Japanese Labor Market

**DOI:** 10.3389/fpsyg.2022.812821

**Published:** 2022-03-15

**Authors:** Satoshi Akutsu, Fumiaki Katsumura, Shohei Yamamoto

**Affiliations:** School of International Corporate Strategy, Hitotsubashi University Business School, Tokyo, Japan

**Keywords:** health management, learning theory, trait activation theory, competitive work environment, subjective health, workaholism, cognitive distortions

## Abstract

The present study examined the direct and indirect (*via* workaholism) relationships between competitive work environments and subjective unhealthiness. It also examined the effects of adjusting for cognitive distortions in the relationship between a competitive work environment and subjective unhealthiness and between a competitive work environment and workaholism. Data were collected from 9,716 workers in various industries, occupations, and positions. The results show that competitive work environments were positively related to subjective unhealthiness, both directly and through workaholism. Furthermore, cognitive distortions moderated the positive effect between a competitive work environment and workaholism, and the positive relationship was stronger when cognitive distortions were high (as compared to low). This study has important and practical implications for companies that are increasingly concerned about the health of their employees.

## Introduction

A decline in employee health represents a major negative factor for individual productivity and the competitiveness of the company as a whole. In particular, mental health problems have become more serious in recent years. The number of people who experience high stress at work is increasing every year, and the amount of insurance payments for mental and behavioral disorders has increased. These problems have been especially serious in Japan. The number has increased fivefold in the past 20 years (National Health Insurance Association, 2017). The suicide rate among depressed patients is said to be about 10%, and the National Institute of Population and Social Security Research (2010) estimates that the economic benefits of eliminating suicide and depression would amount to about 2.7 trillion yen in a single year. In addition, it has been pointed out that corporate losses due to employees who are underperforming because of poor health amount to 7.5% of total labor costs (Collins et al., 2005). It is unfortunate for both the individual worker and the employer that health problems impede work or lead to retirement. Against this background, social interest in promoting employee health has been increasing in Japan, and the Ministry of Economy, Trade and Industry (METI) has taken the lead in promoting “health and productivity management.”

Despite the undisputed importance of each employee’s health to their work productivity and the long-term success of the organization, we are still accumulating theoretical and practical knowledge on how companies can address employee health. To realize the health promotion of workers, this study is based on the literature on the work environment, attitudes toward work, and cognitive traits of workers, which include a competitive work environment (Brown et al., 1998), workaholism (Schaufeli et al., 2008), cognitive distortions (Beck, 1964), and subjective unhealthiness (Sell and Nagpal, 1992). We propose and test a model that integrates these factors.

A competitive work environment is defined as “the degree to which employees perceive organizational rewards to be contingent on comparisons of their performance against that of their peers” (Brown et al., 1998, p. 89). Of course, there are aspects of competition that increase individual motivation and lead to higher performance (Sauers and Bass, 1990). Conversely, the negative effects of competition (psychological load and anti-organizational behavior) have also been highlighted (Fletcher et al., 2008). In particular, several studies have suggested that competitive work environments negatively impact individuals’ subjective health (e.g., Gillespie et al., 2001). However, there are few studies on mediating mechanisms and boundary conditions. Therefore, the first task of this study is to examine the mechanisms and boundary conditions that mediate the relationship between the competitive work environment and subjective unhealthiness and to clarify the actual mechanism.

To construct the model, this study first presents workaholism as a mechanism that mediates the relationship between a competitive work environment and subjective health using learning theory (Skinner, 1974). Workaholism refers to the tendency to work compulsively and excessively hard (Schaufeli et al., 2008). Workaholism is often discussed as a personal trait (e.g., Andreassen et al., 2010; Clark et al., 2010), but its cognitive aspects have also been understood (Beck, 1995). In other words, if an individual believes that hard work is a condition for success in the work environment, they are more likely to engage in workaholic behavior. According to learning theory (Skinner, 1974), individuals learn from the consequences of their past actions to determine whether they should take similar actions in the future. We examine the competitive work environment and its effects on the workaholic behavior tendency. We also discuss and empirically examine how workaholism affects subjective unhealthiness. Indeed, several previous studies such as Wallace (1997) have shown that workaholism negatively impacts individual health.

Moreover, this study shows that cognitive distortion, which is an individual personality trait, acts as a boundary condition for the relationship between a competitive work environment and subjective unhealthiness and between a competitive work environment and workaholism. Tett and Guterman (2000) describe personality traits as intra-individually consistent and inter-individually distinct propensities to behave in some identifiable way. Previous studies have examined the relationship between personality traits and workaholism (Clark et al., 2016; Avanzi et al., 2020; Mazzetti et al., 2020); however, some have shown inconsistent or even seemingly contradicting results for the impact of the same individual trait on workaholism, motivating further examination of the boundary conditions (Clark et al., 2016). According to trait activation theory (Tett and Burnett, 2003), an individual’s personality traits are expressed using the environment in which the individual is placed as a cue. In other words, individuals express their traits based on situational cues that relate to their own traits. Thus, trait activation theory points out that individual traits are enhanced (or mitigated) by interaction with specific environmental factors (Ng et al., 2007). In this study, we focus on cognitive distortion as an individual trait and examine its interaction with a competitive work environment.

**FIGURE 1:**
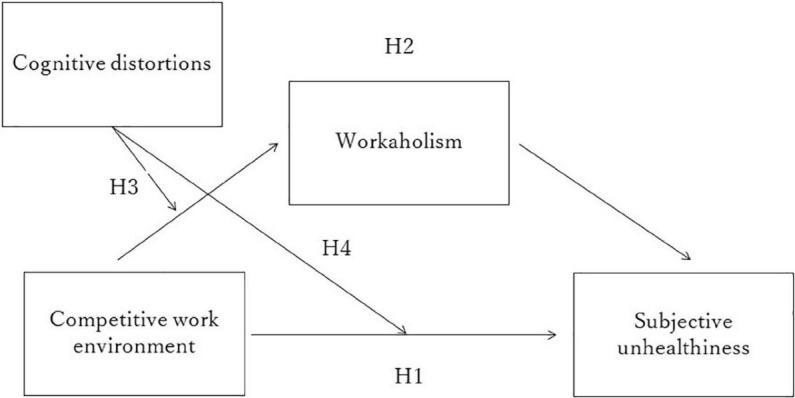
The proposed model (Hypothesis 5).

Cognitive distortion refers to a type of thinking that results in negative automatic thoughts from unrealistic and illogical reasoning (Mikawa, 2004a,b). Cognitive distortion, as an adjustment variable, is important for theory and practice. Individuals with high cognitive distortions tend to have extreme perceptions of their environment and are thus more likely to have an increased sense of duty and obligation toward their work in a competitive work environment. Such cognitive patterns are likely to increase the psychological burden on the self and thus interfere with the health of the individual. Therefore, it is important to understand the effects of cognitive distortions on the relationship between competitive work environments and subjective unhealthiness and between competitive work environments and workaholism. In this study, we argue that the strength of the indirect association between a competitive work environment and subjective unhealthiness *via* workaholism depends on the level of cognitive distortion and then present the adjustment mediation model (see Figure 1 for the proposed model) as a comprehensive psychological mechanism. We believe that by focusing on the role of the adjustment effect of cognitive distortions, it will be possible to explain why differences in individuals’ thinking patterns produce differential effects on workaholism and subjective unhealthiness.

## Theory and Hypotheses

### Competitive Work Environment and Subjective Unhealthiness

Employee health has been a pivotal topic for both researchers and practitioners for more than a century (Loh et al., 2019). In Japan, as many companies are shifting from the traditional seniority-based personnel system to a performance-based and competitive personnel system, the competitive pressure on individual employees is increasing, and their psychological burden is growing. According to a survey conducted by the Ministry of Health, Labour and Welfare, about 60% of workers feel stressed at work (Ministry of Health, Labour and Welfare, 2018). In this study, we empirically examine the relationship between competitive work environment and employee health.

How does competition affect the performance of individuals and organizations? Previous studies have highlighted both positive and negative relationships between competition and performance. From a positive perspective, it has been pointed out that competition with rivals, for example, increases commitment and thus has a positive effect on performance, as in the case of athletes (Lam, 2012). Furthermore, according to social comparison theory (Festinger, 1954), people try to compare themselves with others to affirm their own abilities, and, consequently, competition is more likely to occur in interpersonal relationships (Fletcher et al., 2008). By contrast, from the perspective of negative relationships, it has been argued that competition reduces organizational productivity by encouraging behaviors that undermine the performance of others and by reducing cooperation (Kohn, 1993). Stanne et al. (1999) empirically demonstrated through a meta-analysis that competition does not contribute to the improvement of organizational performance. However, previous studies have seldom discussed the psychological burden of competition on individuals and the physical and mental damage caused by defeat. The few studies that have examined these issues suggest that competitive work environments contribute to the psychological stress of workers and negatively affect their health (Gillespie et al., 2001; Fletcher et al., 2008). For example, it has been pointed out that, even if an individual does their best in a highly competitive environment, their performance will be evaluated in comparison with colleagues, resulting in a high level of uncertainty, which induces stress (Fletcher et al., 2008). In addition, in their discussion of the impact of relationships with coworkers on workers’ mental health, Bronkhorst et al. (2015) suggest that competitive relationships with coworkers in a competitive work environment negatively impact individual health. In light of this discussion of previous studies, it can be inferred that competitive work environments have undesirable effects on individual health, even though they may have desirable effects on performance. Therefore, it is expected that a positive relationship exists between the competitive work environment and employees’ subjective sense of unhealthiness.

Hypothesis 1: A competitive work environment positively affects employees’ subjective unhealthiness.

### Workaholism as a Mediating Variable

Workaholism causes individuals to feel threatened by the need to be engaged in work, sets a close psychological distance from work, and facilitates the act of working long hours as a behavior (Ng et al., 2007). Researchers’ interest in workaholism has grown rapidly in recent years (Clark et al., 2020). Much of the previous research on workaholism (e.g., Taris et al., 2005; Bakker et al., 2009; Gorgievski et al., 2014; Atroszko et al., 2020; Dutheil et al., 2020) has focused on its negative effects and has suggested that these effects should be prevented as much as possible, as they lead to more disadvantages than advantages for both individuals and organizations (Balducci et al., 2020).

Based on learning theory (Skinner, 1974), competitive environments encourage people to perform better than those around them, which increases the likelihood that they will engage in more work than necessary or feel guilty about time spent not working. Furthermore, in a competitive work environment, significant others such as supervisors and coworkers are also expected to produce results, and it is highly likely that they also work in a workaholic manner; observing such behaviors may further reinforce an individual’s tendency toward workaholic behavior. Previous research suggests that when workaholic behavior is affirmed or not punished, individuals understand that workaholism is rewarding and they consequently increase their workaholic behavior for ongoing reward (Ng et al., 2007). In other words, organizational culture and the workplace atmosphere are thought to act as reinforcers of workaholism. For example, Keller et al. (2016) showed that a competitive work environment contributes to workaholism. Furthermore, it has been pointed out that workaholism is positively related to salary increases and promotions in the workplace (Burke, 2001). It has also been highlighted that the globalized competitive environment in which modern companies operate affirms long working hours and reinforces workaholic behavior (Balducci et al., 2020). Based on these findings, it is undeniable that some aspects of workaholic behavior are easily affirmed in many companies. In general, it is expected that a competitive work environment will make people feel that they have to work hard all the time, which in turn will promote workaholic behavior; that is, there is a positive relationship between a competitive work environment and workaholism.

In addition, previous studies have suggested that workaholic behavior negatively affects individual health (Shimazu and Schaufeli, 2009; Clark et al., 2010, 2016; Shimazu et al., 2012; Keller et al., 2016; Atroszko et al., 2020; Dutheil et al., 2020). Workaholism is thought to make people cognitively exhausted over time because there is not enough time (e.g., sleep) and opportunity (e.g., leisure) to recover from excessive work commitment (Taris et al., 2005). Wallace (1997), for example, showed that when work consumes too much of one’s lifetime, it negatively impacts health. Meta-analyses of workaholism have shown that workaholism increases burnout and mental fatigue (Clark et al., 2016). Other studies have shown that workaholics have poorer physical health than non-workaholics because they have less leisure time and exercise (Kanai et al., 1996; Ng et al., 2007). Furthermore, workaholics are more likely to work long hours because they have certain addictions and obsessions with work, and a meta-analysis by Sparks et al. (1997) showed that long working hours is associated with many physical ailments. Therefore, workaholism is expected to be positively related to subjective feelings of unhealthiness. From the above discussion, the following hypothesis can be derived:

Hypothesis 2: Workaholism mediates the positive impact of a competitive work environment on subjective unhealthiness.

### Cognitive Distortion as a Moderator

Cognitive distortions are distorted perceptions of a situation, errors in reasoning, and thought patterns that lead to a negative view of a situation (Beck, 1964; Mikawa, 2004a,b). In their research on depressed patients, Beck et al. (1979) pointed out that such patients tend to form negative automatic thoughts from unrealistic and illogical reasoning in situations in which they experience negative events. Trait activation theory (Tett and Guterman, 2000; Tett and Burnett, 2003) refers to the situational specificity of personal characteristics and job performance. The basis for this theory is that the interaction between environmental factors and the expression of an individual’s personality is based on certain situational cues. There have been many studies on the relationship between individual personality and job performance (e.g., Barrick and Mount, 1991; Hough, 1992). The results of these studies have revealed that the relationship between personality traits and job performance can be predicted to some extent but can also vary depending on the specific situation (Tett and Burnett, 2003). An important aspect of trait activation theory proposed in this problematic context is that it focuses on the trait relevance of a situation and its strength (Tett and Guterman, 2000), meaning that a given personal trait will be reflected in an individual’s behavior depending on the situation in which it is activated and on its intensity.

The competitive work environment tends to encourage overtime work because it focuses workers’ attention on work performance. In addition, workers are expected to feel guilty about being distant from their work, even outside of working hours. It is not hard to imagine that in such an environment, the personal trait of cognitive distortion is activated. Individuals with high cognitive distortions are more likely to amplify the negative aspects of a competitive work environment and to feel overcommitted, responsible, or obligated. According to Mikawa (2004a,b), cognitive distortion consists of three components: “cognitive bias,” “reasoning errors,” and “inflexibility of thought.” “Cognitive bias” refers to the tendency to consider everything in terms of two choices and to exclude any in-between options or ambiguity (bipartite thinking). “Reasoning error” refers to the tendency to create generalized rules from fragmentary facts and events (overgeneralization), whereas “inflexibility of thought” means to think in absolutes, such as “I must” or “I should” (definitive thinking). Since competitive environments tend to encourage compulsive and excessive work, cognitive distortions are expected to amplify this tendency. In such a work environment, individuals with high cognitive distortions are likely to be hesitant to come to terms with their work and take a break, and they are likely to be overly concerned about adapting themselves to the standards expected by such a work environment. As a result, they are expected to drive themselves toward more workaholic behavior. Based on this line of reasoning, we would expect that individuals with high cognitive distortions would be more likely to amplify the workaholic behavioral tendencies brought about by a competitive work environment.

Hypothesis 3: Cognitive distortions moderate the positive impact of competitive work environments on workaholism, and the relationship is stronger when cognitive distortions are high.

In addition, people are more likely to feel anxious and depressed when experiencing distress, such as psychological challenges and obstacles (Ingram, 1990). Beck et al. (1979) described the negative automatic thoughts that result from unrealistic and illogical reasoning when experiencing negative events as a characteristic of cognitive distortions. In other words, it can be inferred that individuals with high cognitive distortion tend to be more biased toward the negative aspects of a particular situation and, as a result, are more likely to feel the psychological load and experience anxiety. These inferences suggest that individuals with high cognitive distortions are more likely than individuals with low cognitive distortions to have automatic thoughts about the negative aspects of a particular environment and, as a result, are more likely to experience negative health effects. Therefore, we expect that individuals with high cognitive distortions would exhibit a stronger positive relationship between competitive work environments and subjective unhealthiness than individuals with low cognitive distortions.

Hypothesis 4: Cognitive distortions moderate the positive impact of competitive work environments on subjective unhealthiness, and this relationship is stronger when cognitive distortions are high.

As discussed above, high levels of cognitive distortion amplify the positive effect of a competitive work environment on employees’ workaholism. Therefore, the mediating effect of workaholism on the positive relationship between a competitive work environment and subjective unhealthiness is expected to depend on the level of cognitive distortion. From a statistical perspective, the authors present a moderated mediation model. In other words, the extent to which workaholism (the mediating variable) mediates the effect of a competitive work environment (the independent variable) on subjective unhealthiness (the dependent variable) is expected to depend on the level of cognitive distortion (the moderator).

While we argue above that cognitive distortion moderates the impact of a competitive environment on workaholism as well as the impact of a competitive environment on subjective unhealthiness, we do not mean that cognitive distortion moderates the impact of workaholism on subjective unhealthiness. As we argue, workaholism is considered to affect subjective unhealthiness primarily through its accompanying behavior (i.e., workaholic behavior). Given that subjective unhealthiness is a behavioral consequence of workaholism, cognitive distortion may not moderate their relationship. Rather, it may affect workaholism in conjunction with a competitive work environment. As such, we formulate the following hypothesis:

Hypothesis 5: The indirect relationship between competitive work environment and subjective unhealthiness *via* workaholism is moderated by cognitive distortion. The indirect effect is stronger for people with higher cognitive distortions (compared to those with lower cognitive distortions).

## Research Method

### Data Overview

This study used data from an online survey of individuals’ job satisfaction and lively working conditions conducted by the Recruit Works Institute from November 13 to 15, 2019. The survey participants were recruited on a voluntary basis, and their privacy was protected by the institute’s ethical protocol following the Japanese Personal Information Protection Law.

The survey was based on the “Labor Force Survey” of the Statistics Bureau of Japan and the educational background data of the Ministry of Education, Culture, Sports, Science and Technology (MEXT). The respondents were prescreened to select participants with attributes (gender, age group, employment status, regional block, and educational background) with the same distribution ratio as the population.

### Sample

A total of 9,716 people responded to the survey: 51% male (4,961), age range 18–89 years (Mage 46; SDage 13.66); occupations included mining (0.1%), agriculture, forestry, and fishing (0.9%); water supply (1.6%); real estate (2.6%); restaurants and lodging (2.9%); financial and insurance (3.2%); education and learning support (3.8%); public services (4.1%); electricity, gas, heat supply, and information and communication (4.6%); transportation (5.2%); construction (6.4%); wholesale and retail (11.6%); healthcare and welfare (12.1%); services (15.8%); manufacturing (16.7%); and none of the above (8.3%). Their highest level of education corresponded to doctorate (0.7%), elementary or junior high school (2.5%); master’s degree (2.6%); bachelor’s degree (25.8%); professional training college, vocational school, junior college, or technology college (30.6%); and high school (37.7%).

### Measurement

#### Competitive Work Environment

The survey included three items from the scale developed by Sekimoto et al. (2001), and the mean value of the scale was used as the variable for the competitive work environment in this study. Specifically, the items were: “If I do not achieve good results in terms of work, I have to look over my shoulder”; “There is a place where those who win the competition are rewarded accordingly”; and “No matter how hard I work, if the results are not good, there is a place where I am not taken seriously.” These questions were answered using a six-point scale ranging from “1 = it does not describe me at all” to “6 = it perfectly describes me.” Cronbach’s alpha was 0.762 in this study.

#### Subjective Unhealthiness

The mean values of the six items constituting the “perception of unhealthiness” factor from the Japanese version of Sell and Nagpal (1992) Subjective Well-Being Inventory (SUBI) (Tonan et al., 1995) were used as variables of subjective unhealthiness in this study. The specific items were: “Do you sometimes worry about your health?”; “Do you suffer from pain in various parts of your body?”; “Are you disturbed by palpitations/a thumping heart?”; “Are you disturbed by a feeling of giddiness?”; “Do you feel you get tired too easily?”; and “Are you troubled by disturbed sleep?” These questions were answered on a three-point scale ranging from “1 = not so much” to “3 = always.” Cronbach’s alpha was 0.794 in this study.

#### Workaholism

Based on Schaufeli et al.’s (2009) survey on work and wellbeing (DUWAS), we measured “Workaholism.” The Schaufeli et al. (2009) scale consists of 10 questions, five of which measure working excessively (WE), while the remaining five measure working compulsively (WC). In the present study, the mean of six items, three from each, was used as the variable for workaholism. Specifically, “I seem to be in a hurry and racing against the clock” (WE); “I find myself continuing to work after my coworkers have called it quits” (WE); “I stay busy and keep many irons in the fire” (WE); “It is important to me to work hard even when I do not enjoy what I am doing” (WC); “I feel that there is something inside me that drives me to work hard” (WC); and “I feel obliged to work hard, even when it is not enjoyable” (WC). These questions were answered using a four-point scale ranging from “1 = (almost) never” to “4 = (almost) always.” The exploratory factor analysis of these six items resulted in one factor. Moreover, the reliability analysis showed that Cronbach’s alpha was WE = 0.780 and WC = 0.804. Cronbach’s alpha for the six items was 0.857. Therefore, in this study, the sum of the WE and WC items was used as the workaholism variable in the analysis.

#### Cognitive Distortion

Three factors of the cognitive distortion scale developed by Azuma (1996) were selected for data collection: “split-thinking”; “categorical expression”; and “overgeneralization,” and the combined average of these three factors was used as the cognitive distortion variable in this study. The mean value was used as the “cognitive distortion” variable. In concrete terms, “I think that things are either worthwhile or not worthwhile” (bipartite thinking); “I think that life is either a success or a failure” (bipartite thinking); “Judging people’s actions as ‘good’ or ‘bad”’ (bipartite thinking); “I often say ‘completely’ or ‘absolutely”’ (definitive expressions); “I often say, ‘I must’ or ‘I should not”’ (definite expression); “I often say, ‘It is only natural’ or ‘Of course it is”’ (definite expression); “I feel that if I fail at one thing, everything will go wrong” (overgeneralization); “If people don’t like me, I feel like no one will like me anymore” (overgeneralization); and “I feel that if I have one bad thing, everything in the world will be bad” (overgeneralization). These questions were answered on a five-point scale from “1 = it does not describe me at all” to “5 = it perfectly describes me.” Cronbach’s alpha was 0.824 in this study.

#### Control Variables

As the control variables, we first included typical sociodemographic variables: age, gender (1 = male; 2 = female), marital status (1 = married; 2 = single), education (1 = elementary school or junior high school to 8 = doctoral degree), and annual household income. These sociodemographic factors can be considered standard control variables for both workaholism and subjective unhealthiness.

Workaholism appears to be correlated with some job-related variables such as job position (e.g., Kanai and Wakabayashi, 2001; Ng and Feldman, 2008). This finding was also confirmed in a meta-analysis (Clark et al., 2016). Thus, we included the following job-related variables: company size (i.e., number of employees) and position (1 = top management to 8 = rank-and-file). The relationships between workaholism and non-work-related activities have also been actively studied (e.g., Kanai et al., 1996; Kubota et al., 2014). More specifically, some studies have shown that lifestyle outside of work and family influences individuals’ workaholism and health (e.g., Kanai et al., 1996; Wallace, 1997; Taris et al., 2005; Britt et al., 2013; Kubota et al., 2014; Dutheil et al., 2020; Shimazu et al., 2020). Therefore, we added a variable to control for non-work activity level by asking the degree to which individuals perform “arts, hobbies, and sports activities.” Similarly, we added a set of variables to control for family-related activity level by asking the degree to which individuals do the following: “activities to keep family in a good condition,” “eating with family,” “talking with family for more than 30 minutes,” and “going somewhere with family.” Moreover, we added a set of variables to control for lifestyle habits by asking the degree to which individuals “eat breakfast,” “get enough sleep,” “consume alcohol,” and “smoke.” All questions were measured using a seven-point scale [1 = never; 7 = always (almost every day)].

Prior research has suggested a general relationship exists between workaholism and organizational climate (e.g., Johnstone and Johnston, 2005; Keller et al., 2016). In this study, we focused on competitive organizational climate and hypothesized that it promotes workaholism. To improve rigor, we tested this hypothesis after controlling for the effects of other types of organizational climate. Thus, we included all six factors for organizational climate used by Sekimoto et al. (2001), from whose research we obtained the “competitive work environment” variable for this study. Each factor for competitive work environment consisted of three items, except for respect for the individual, which consisted of six items. The six factors were authoritarianism/avoidance of responsibility (Cronbach’s alpha = 0.763); free and open-mindedness (Cronbach’s alpha = 0.800); long-term and big-picture orientation (Cronbach’s alpha = 0.818); flexibility, creativity, and originality (Cronbach’s alpha = 0.837); prudence and meticulousness (Cronbach’s alpha = 0.743); and respect for the individual (Cronbach’s alpha = 0.890). Since the Cronbach’s alpha for the six factors was acceptable (c.f., Pallant, 2011), we included all of them as control variable in our analyses.

Lastly, regarding individual characteristics related to workaholism, self-esteem, recognition of strengths, resilience, work meaningfulness, psychological safety, and the Big Five have been discussed in relevant literature (e.g., Barrick and Mount, 1991; Burke et al., 2006; Graves et al., 2012; Clark et al., 2016; Gillet et al., 2017; Choi et al., 2020; Kasemy et al., 2020; Kun et al., 2020). As their corresponding measurements were available in the dataset we used, we considered the possibility of including them as control variable in our analyses. Specifically, the dataset had two items from Minoura and Narita’s (2013) Self-esteem Scale (Cronbach’s alpha = NA), four items from Takahashi and Morimoto (2015) Recognition of Strengths Scale (Cronbach’s alpha = 0.842), three items for each of the four factors from Hirano’s (2010) Resilience Scale [optimism (Cronbach’s alpha = 0.841), control (Cronbach’s alpha = 0.674), sociability (Cronbach’s alpha = 0.856), and action (Cronbach’s alpha = 0.793)], nine items from Masaki’s (2016) Work Meaningfulness Scale (Cronbach’s alpha = 0.911), seven items from Maruyama and Fuji’s (2019) Psychological Safety Scale (Cronbach’s alpha = 0.644), and two items for each of the five factors from Oshio et al.’s (2012) Big Five Scale (Cronbach’s alpha = NA). The Cronbach’s alphas for all the variables above, except for self-esteem and the Big Five, where Cronbach’s alpha was not appropriate for the reliability estimate, were acceptable (c.f., Pallant, 2011). Thus, we included them as control variables in our analyses. Since only two items measured self-esteem and the Big Five factors in the dataset, there was a conceptual concern because the psychological latent constructs were measured by only one or two items (e.g., Eisinga et al., 2013). Therefore, it is suggested to use the Spearman–Brown formula (vs. Cronbach’s alpha) to examine their reliability (Eisinga et al., 2013). The Spearman–Brown reliability estimate for self-esteem was 0.5, which is out of the acceptable range (0.2–0.4) suggested by Briggs and Cheek (1986). For the Big Five, the Spearman–Brown reliability estimates for four factors were much smaller than the minimum acceptable value of .2 (i.e., 0.117 for agreeableness, 0.056 for conscientiousness, 0.168 for neuroticism, and 0.100 for openness). Thus, due to both conceptual and empirical concerns, we decided not to include self-esteem and the Big Five as control variable in our analyses. While we did our best to include relevant control variables in this study, it should be noted that the choice of control variables was restricted by the availability in the Recruit Works Institute dataset that we used.

### Data Analysis and Procedure

To examine our hypotheses, we executed the following procedure for data analysis. First, we calculated basic descriptive statistics for the key variables. Second, we tested Hypotheses 1 and 2 using Hayes’ PROCESS macro, Model 4 (Hayes, 2017). Third, we tested Hypotheses 3, 4, and 5 with Hayes’ PROCESS macro, Model 8 (Hayes, 2017). We used SPSS version 26.0 and PROCESS macro version 2.13.

## Results

### Means and Correlations

The mean values and correlations of the variables in this study are presented in Table 1. Note that since we have quite a few control variables in our analyses (=29), we excluded them from Table 1. The means, standard deviations, and correlations of the control variables are available in the Supplementary Appendix.

**TABLE 1:** Means and correlations.

Construct	Means	SD	1	2	3
(1) Competitive work environment	2.856	0.746			
(2) Workaholism	2.014	0.694	0.253**		
(3) Subjective unhealthiness	1.565	0.448	0.137**	0.309**	
(4) Cognitive distortion	2.660	0.617	0.271**	0.232**	0.261**

N = 9,716. *p < 0.05 and **p < 0.01 level (two-tailed). SD, standard deviation.

### Results of Mediation Analysis

Hayes’ PROCESS macro, Model 4 (Hayes, 2017) was used to test Hypotheses 1 and 2. First, the direct effect from competitive work environment to subjective unhealthiness was significant (β = 0.032, *t* = 4.347, *p* < 0.001). Thus, Hypothesis 1 is supported. When “workaholism” was incorporated as a mediator, however, the direct effect became weaker (β = 0.014, *t* = 1.982, *p* < 0.05). The indirect effect was significant at 0.018 (95% confidence interval (CI) did not include zero, 0.014–0.022). Thus, Hypothesis 2 is supported. Table 2 summarizes the results. It should be noted that we included all the aforementioned control variables in this analysis as well as the following analysis.

**TABLE 2:** Mediation results.

	Bootstrapped CI 95%						
	β	SE	*t*	*p*	LL	UL	*R* ^2^
	
Model 1: Mediator variable model	Outcome: Workaholism						
Constant	1.425	0.079	18.003	0.000	1.269	1.580	0.164
Competitive work environment	0.101	0.011	9.202	0.000	0.079	0.122	
Age	–0.006	0.001	–11.545	0.000	–0.007	–0.005	
Gender (1 = male; 2 = female)	0.024	0.014	1.698	0.090	–0.004	0.053	
Marital status (1 = married; 2 = single)	–0.016	0.016	–1.004	0.316	–0.047	0.015	
Education	–0.009	0.004	–2.436	0.015	–0.017	–0.002	
Annual household income	0.000	0.000	0.179	0.858	0.000	0.000	
Company size	0.007	0.002	3.562	0.000	0.003	0.011	
Position	–0.012	0.003	–3.947	0.000	–0.018	–0.006	
Arts, hobbies, and sports activities	0.001	0.004	0.256	0.798	–0.006	0.008	
Activities to keep family in a good condition	0.013	0.004	3.469	0.001	0.006	0.020	
Eating with family	–0.015	0.006	–2.699	0.007	–0.026	–0.004	
Talking with family for more than 30 min	–0.003	0.006	–0.531	0.595	–0.015	0.009	
Going somewhere with family	0.035	0.006	6.211	0.000	0.024	0.046	
Eating breakfast	–0.001	0.004	–0.318	0.751	–0.008	0.006	
Getting enough sleep	–0.048	0.004	–12.051	0.000	–0.056	–0.040	
Alcohol consumption	–0.001	0.003	–0.306	0.760	–0.007	0.005	
Smoking	0.012	0.003	4.322	0.000	0.007	0.018	
Authoritarianism/avoidance of responsibility	0.128	0.010	13.132	0.000	0.109	0.147	
Free and open-mindedness	–0.003	0.013	–0.216	0.829	–0.029	0.023	
Long-term and big-picture orientation	0.025	0.015	1.746	0.081	–0.003	0.054	
Flexibility, creativity and originality	0.040	0.014	2.882	0.004	0.013	0.067	
Prudence and meticulousness	0.029	0.014	2.095	0.036	0.002	0.055	
Respect for the individual	0.006	0.016	0.376	0.707	–0.026	0.038	
Recognition of strengths	–0.009	0.013	–0.696	0.487	–0.035	0.017	
Resilience (optimism)	–0.062	0.012	–5.288	0.000	–0.085	–0.039	
Resilience (control)	0.033	0.013	2.627	0.009	0.008	0.058	
Resilience (sociability)	0.000	0.009	0.033	0.974	–0.018	0.019	
Resilience (action)	0.113	0.012	9.237	0.000	0.089	0.137	
Work meaningfulness	0.087	0.008	10.535	0.000	0.071	0.104	
Psychological safety	–0.117	0.013	–8.954	0.000	–0.143	–0.092	

**Model 2: Outcome variable model**	**Outcome: Subjective unhealthiness**						

Constant	1.539	0.052	29.751	0.000	1.438	1.641	0.170
Workaholism	0.176	0.007	26.912	0.000	0.163	0.189	
Competitive work environment	0.014	0.007	1.982	0.048	0.000	0.028	
Age	0.000	0.000	0.340	0.734	–0.001	0.001	
Gender (1 = male; 2 = female)	0.023	0.009	2.506	0.012	0.005	0.041	
Marital status (1 = married; 2 = single)	0.045	0.010	4.431	0.000	0.025	0.065	
Education	–0.009	0.002	–3.658	0.000	–0.014	–0.004	
Annual household income	0.000	0.000	–2.253	0.024	0.000	0.000	
Company size	–0.002	0.001	–1.317	0.188	–0.004	0.001	
Position	–0.004	0.002	–2.155	0.031	–0.008	0.000	
Arts, hobbies, and sports activities	0.004	0.002	1.585	0.113	–0.001	0.009	
Activities to keep family in good condition	0.002	0.002	0.630	0.529	–0.003	0.006	
Eating with family	–0.005	0.004	–1.393	0.164	–0.012	0.002	
Talking with family for more than 30 min	–0.006	0.004	–1.532	0.126	–0.013	0.002	
Going somewhere with family	0.024	0.004	6.637	0.000	0.017	0.031	
Eating breakfast	0.001	0.002	0.540	0.589	–0.003	0.006	
Getting enough sleep	–0.037	0.003	–14.446	0.000	–0.042	–0.032	
Alcohol consumption	0.004	0.002	1.787	0.074	0.000	0.007	
Smoking	0.005	0.002	2.920	0.004	0.002	0.009	
Authoritarianism/avoidance of responsibility	0.017	0.006	2.748	0.006	0.005	0.030	
Free and open-mindedness	0.012	0.008	1.378	0.168	–0.005	0.028	
Long-term and big-picture orientation	0.000	0.009	–0.021	0.984	–0.018	0.018	
Flexibility, creativity and originality	0.012	0.009	1.377	0.169	–0.005	0.030	
Prudence and meticulousness	0.002	0.009	0.228	0.820	–0.015	0.019	
Respect for the individual	0.016	0.010	1.514	0.130	–0.005	0.036	
Recognition of strengths	0.043	0.009	5.016	0.000	0.026	0.059	
Resilience (optimism)	–0.021	0.008	–2.817	0.005	–0.036	–0.006	
Resilience (control)	–0.101	0.008	–12.443	0.000	–0.117	–0.085	
Resilience (sociability)	0.020	0.006	3.282	0.001	0.008	0.032	
Resilience (action)	0.013	0.008	1.677	0.094	–0.002	0.029	
Work meaningfulness	–0.024	0.005	–4.544	0.000	–0.035	–0.014	
Psychological safety	–0.060	0.008	–7.075	0.000	–0.076	–0.043	
**Bootstrapping results for the indirect effect**							
The indirect effects of Workaholism on the impact of competitive work environments on subjective unhealthiness.	0.018	0.002			0.014	0.022	

*N = 9,716, Bootstrap sample size = 1,000. β, unstandardized regression coefficients; LL, lower limit; CI, confidence interval; UL, upper limit.*

As shown in Table 2, the following control variables were significantly associated with workaholism and/or subjective unhealthiness. First, for sociodemographic variables, age and education were found to be significantly associated with workaholism. More specifically, the older and more highly educated, the lower the workaholism level. Similarly, for subjective unhealthiness, gender, marital status, education, and annual household income were found to be significantly associated. More specifically, being female (vs. male) and single (vs. married) showed higher subjective unhealthiness. Additionally, with a higher education level and higher annual household income, subjective unhealthiness was lower.

For job-related variables, both company size and position had a significant association with workaholism. Specifically, the larger the company and the higher the job position, the higher the workaholism level. Position also had a significant association with subjective unhealthiness, with higher job position being related to higher subjective unhealthiness. Activity level outside of work had a significant relationship with either workaholism or subjective unhealthiness. For family-related activity level, activities to keep family in a good condition and going somewhere with family were significantly positively related to workaholism. One possible reason for this could be that workaholic workers may feel guilty for neglecting their family and try to compensate by consciously focusing on them. Interestingly, in contrast, eating with family, which can be considered a part of lifestyle (vs. compensation), had a negative relationship with workaholism. For other lifestyle habits, getting enough sleep had a negative relationship with both workaholism and subjective unhealthiness, whereas smoking had a positive relationship with both, which is expected.

Regarding factors related to organizational climate, authoritarianism/avoidance of responsibility; flexibility, creativity, and originality; and prudence and meticulousness had a significant positive relationship with workaholism. It is possible that authoritarianism/avoidance of responsibility and prudence and meticulousness positively relate to workaholism through work demands and pressure and that flexibility, creativity, and originality positively relate to workaholism through intrinsic work motivation. Authoritarianism/avoidance of responsibility was also significantly positively related to subjective unhealthiness.

Lastly, for individual characteristics, resilience (optimism) and psychological safety had a significant negative relationship with workaholism, whereas resilience (control), resilience (action), and work meaningfulness had a significant positive relationship with workaholism. It should be noted that resilience (control) and resilience (action) are associated with a capacity for endurance and perseverance, respectively, which may induce workaholic thoughts and behavior. Interestingly, resilience (optimism), resilience (control), work meaningfulness, and psychological safety had a significant negative relationship with subjective unhealthiness versus a significant positive relationship for recognition of strength and resilience (sociability). For resilience (control) and work meaningfulness, the relational direction with workaholism was opposite to that with subjective unhealthiness, suggesting complicated mechanisms.

### Results of the Moderation Analysis

Hayes’ PROCESS macro, Model 8 (Hayes, 2017) was used to test the adjustment effect of cognitive distortions on the relationship between competitive work environment and workaholism (Hypothesis 3), the adjustment effect of cognitive distortions on the relationship between competitive work environment and subjective unhealthiness (Hypothesis 4), and the adjustment effect of cognitive distortions on the indirect relationship between competitive work environment and subjective unhealthiness (*via* workaholism) (Hypothesis 5). Since this study aimed to examine the moderating effect of cognitive distortion on the direct relationship between competitive work environment and workaholism, the relationship between competitive work environment and subjective unhealthiness, and the indirect relationship between competitive work environment and subjective unhealthiness (*via* workaholism), Model 8 was used to test all of these hypotheses simultaneously.

**FIGURE 2:**
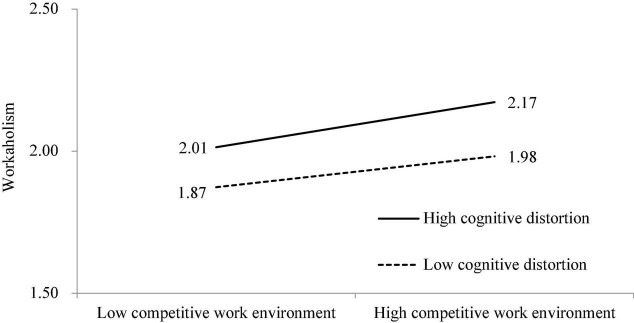
The moderating effect of cognitive distortions on the relationship between competitive work environment and workaholism.

First, for Hypothesis 3, the effect of the interaction term between a competitive work environment and cognitive distortion on workaholism was significant (β = 0.027, *p* < 0.05), indicating that cognitive distortion moderates the positive effect of competitive work environment and workaholism. A simple slope test was conducted to examine the strength of the relationship between competitive work environment and workaholism at high and low levels of cognitive distortion. The results show that the positive relationship between competitive work environment and workaholism was greater when cognitive distortion was high (β = 0.106, *t* = 8.173, *p* < 0.001) than when it was low (β = 0.073, *t* = 5.863, *p* < 0.001) (Figure 2). Therefore, Hypothesis 3 is supported.

**FIGURE 3:**
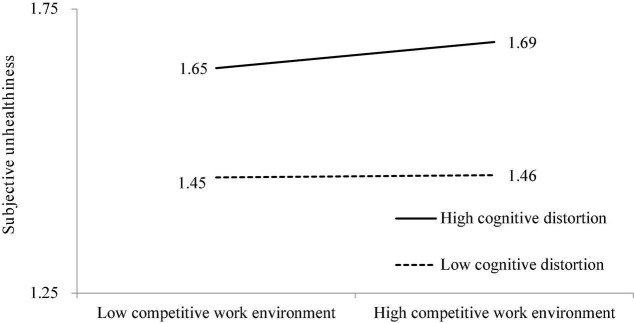
The moderating effect of cognitive distortions on the relationship between competitive work environment and subjective unhealthiness.

As for Hypothesis 4, the moderating effect of cognitive distortion on competitive work environment and subjective unhealthiness was significant (β = 0.023, *p* < 0.01). A simple slope test was conducted to examine the strength of the relationship between competitive work environment and subjective unhealthiness at high and low levels of cognitive distortion. The results show that the effect of competitive work environment on subjective unhealthiness was significant when cognitive distortion was high (β = 0.031, *t* = 3.627, *p* < 0.001), but not when it was low (β = 0.003, *t* = 0.350, n.s.) (Figure 3). Thus, Hypothesis 4 is supported.

### The Result of the Moderated Mediation Model

Finally, for Hypothesis 5, cognitive distortion was shown to moderate the mediating effect of workaholism on the relationship between competitive work environment and subjective unhealthiness (Hayes’ moderated mediation model index = 0.004, SE = 0.002, 95% CI: 0.001–0.008). As shown in Table 3, the mediating effect of workaholism on the relationship between competitive work environment and subjective unhealthiness was greater when cognitive distortion was high (compared to when it was low). Thus, Hypothesis 5 is supported.

**TABLE 3:** The result of the moderated mediation model.

	Bootstrapped CI 95%						
	β	SE	*t*	*p*	LL	UL	*R* ^2^
	
Model 1: mediator variable model	Outcome: Workaholism						
Competitive work environment	0.090	0.011	8.206	0.000	0.068	0.111	0.175
Cognitive distortion	0.134	0.012	11.480	0.000	0.111	0.157	
Competitive work environment × Cognitive distortion	0.027	0.011	2.575	0.010	0.007	0.048	
**The conditional direct effects of competitive work environments on Workaholism**							
Cognitive distortion (−1 SD)	0.073	0.012	5.863	0.000	0.049	0.097	
Cognitive distortion (+1 SD)	0.106	0.013	8.173	0.000	0.081	0.132	

**Model 2: outcome variable model**	**Outcome: Subjective unhealthiness**						

Competitive work environment	0.003	0.007	0.359	0.720	–0.011	0.016	0.204
Workaholism	0.161	0.006	24.896	0.000	0.148	0.173	
Cognitive distortion	0.152	0.008	20.272	0.000	0.137	0.166	
Competitive work environment × Cognitive distortion	0.018	0.007	2.735	0.006	0.005	0.032	
**The conditional direct effects of competitive work environments on subjective unhealthiness**							
Cognitive distortion (−1 SD)	–0.009	0.008	–1.120	0.263	–0.024	0.007	
Cognitive distortion (+1 SD)	0.014	0.008	1.668	0.095	–0.002	0.030	
**Bootstrapping result of indirect effect (*via* Workaholism)**							
Index of moderated mediation	0.004	0.002			0.001	0.008	
**The conditional indirect effect of competitive work environments on subjective unhealthiness (*via* Workaholism)**							
Cognitive distortion (−1 SD)	0.012	0.002			0.008	0.016	
Cognitive distortion (+1 SD)	0.017	0.002			0.013	0.022	

*N = 9,716, Bootstrap sample size = 1,000. β, unstandardized regression coefficients; LL, lower limit; CI, confidence interval; UL, upper limit.*

## Discussion

In light of the negative impact of unhealthiness among workers on their own quality of life, organizational productivity of firms, and government spending on health care, this study focused on the competitive work environment as a factor and empirically examined its positive impact on subjective unhealthiness, using rich data representing general Japanese workers. We found that workaholism mediated the positive causal relationship between a competitive work environment and subjective unhealthiness. More importantly, we found that cognitive distortion moderated the positive effect of a competitive work environment on subjective unhealthiness and that the relationship was stronger when cognitive distortion was higher. Similarly, cognitive distortion moderated the positive effect between the competitive work environment and subjective unhealthiness, and the relationship was strengthened when cognitive distortion was high. Finally, cognitive distortion also moderated the indirect relationship between a competitive work environment and subjective unhealthiness *via* workaholism, with the indirect effect being strengthened when cognitive distortion was high.

The present study advances our understanding of the mechanisms by which competitive work environments lead to subjective unhealthiness. Specifically, we present workaholism as an important mediating mechanism that explains why competitive work environments are positively related to subjective unhealthiness. The present study, based on learning theory, suggests that competitive work environments promote workaholism, which is characterized by excessive and obsessive work behaviors among workers, and that this negatively impacts their health. Although previous studies have focused on organizational climate in relation to worker health (e.g., Becher et al., 2018), few studies have empirically examined competitive work environments. The present study provides a new perspective on the relationship between organizational climate and worker health. It deepens our knowledge of the psychological mechanisms that explain why there is a positive relationship between competitive work environment and subjective unhealthiness by focusing on individuals’ cognitive processes.

In addition, based on trait activation theory, the present study revealed that cognitive distortion functions as a boundary condition in the direct and indirect relationships between competitive work environments and subjective unhealthiness and between competitive work environments and workaholism. The results suggest that environmental factors such as competitive environment and individual characteristics such as cognitive distortion may reinforce each other. Cognitive distortions give rise to negative automatic thoughts from unrealistic and illogical reasoning about the situation (Mikawa, 2004a,b), and such conditions significantly amplify the negative aspects caused by competitive work environments. In other words, cognitive distortions may reinforce the positive relationship between competitive work environments and workaholism and between competitive work environments and subjective unhealthiness. Research on the boundary conditions of workaholism is a topic of growing interest in recent years, but only a few studies (e.g., Falco et al., 2020; Li et al., 2020; Spagnoli et al., 2020) have empirically examined these conditions. The current study empirically examined how individual workers responded to organizational climate at different levels depending on the degree of cognitive distortion, resulting in different degrees of influence of the competitive work environment on workers’ workaholism and subjective unhealthiness. As such, the authors explain why the learning process affects different employees differently and why some employees respond more strongly to a competitive organizational climate than others.

### Practical Implications

This study provides insights into how organizations can improve employee health. It focused on workaholism as a psychological mechanism by which competitive work environments in organizations promote poor health among their members. Whereas a competitive and performance-based work environment in an organization can contribute to enhancing organizational competitiveness, the cognitive behavior of organizational members trying to adapt to such an organizational environment promotes workaholic behavioral tendencies, which in turn negatively impacts their health. In this study, we empirically examined the effects of employees’ cognitive behavior on their health. In addition, the loss of business due to a health-related decrease in individual productivity, although employees are still engaged in their daily work, is considered to seriously affect the competitiveness of the company.

How can we balance employee morale with health? For example, the promotion of work engagement is considered an effective measure. This concept emerged from discussions in industrial psychology and management organization theory and is at the core of a theory called the “job demands-resources” model. Work engagement is a positive and fulfilling work-related psychological state, characterized by vitality (high levels of energy and psychological resilience during work), enthusiasm (strong involvement, enthusiasm, and pride in work), and immersion (concentration and immersion in work) (Schaufeli et al., 2002; Schaufeli and Bakker, 2004; Shimazu and Toyama, 2019). People with high work engagement say that they feel satisfied, enthusiastic, and energized by their work (Shimazu, 2010). According to Shimazu and Toyama (2019), workaholism is the cognition of “I have to work,” while work engagement is the cognition of “I want to work,” and the two are different concepts. Thus, it is important for companies to distinguish between workaholism and work engagement and to promote efforts to suppress workaholism and promote work engagement. Furthermore, work engagement has been shown to have a positive effect on employee health (Shimazu and Schaufeli, 2009). In future research, it is important to examine the antecedents of work engagement. For example, the importance of the meaning of work has been pointed out in improving work engagement; it has been suggested that when a company’s ideals resonate with the values of individual employees, the work engagement of individual employees improves, which in turn leads to enhanced organizational competitiveness (Akutsu and Katsumura, 2016). In research on organizational identification (Ashforth and Mael, 1989; Mael and Ashforth, 1992; Takao, 2015), it has been shown that unity with the organization contributes to the supportive behavior of organizational members toward the organization. In Japan, where expectations for initiatives such as “health management” and “work style reform” are increasing, companies are required more than ever to foster an organizational culture that promotes work engagement. Through such efforts, companies are expected to enhance their individual situational awareness and actively promote efforts to improve work engagement, which will lead to healthy corporate competitiveness.

Furthermore, efforts to correct cognitive distortions have been considered significant. Cognitive distortions are thought to be individual habits of thought that can be improved through education and training. Efforts to correct cognitive distortions have been shown as effective in cognitive-behavioral therapy for depressed patients (Beck et al., 1979; Freeman, 1989; Sakano, 1995). It is important for companies to pay attention to employees with high cognitive distortions and encourage them to correct such thinking by actively adopting human resource systems that mitigate the negative aspects of the competitive environment for employees. Leadership styles that enhance employees’ self-efficacy (Burns, 1978; Bass, 1985; Ishikawa, 2013) are also important. Recent studies have shown that supervisors’ workaholism exhausts their subordinates and raises their turnover intentions (Kim et al., 2020). Since negative effects of workaholism may affect not only individual supervisors but also their subordinates, more attention needs to be paid to the negative effects of workaholism at the team level.

### Limitations and Future Directions

We used survey data from a general Japanese sample. The sample in this study covers a variety of industries, occupations, ages, and positions. Thus, our results are highly generalizable. However, there are several limitations to this study that should be considered when interpreting the results. First, our correlational analysis is limited by the use of cross-sectional data. To ensure causal analysis, we need to use longitudinal survey data or experimental study designs. Given the opportunity to conduct a longitudinal survey in the future, it would be effective to measure the independent variable (Time 1), the mediating variable (Time 2), and the dependent variable (Time 3) at different points in time. Second, although the workaholism scale (Schaufeli et al., 2009) originally consists of 10 items, in this study, it was measured with six items (three WE items and three WC items) due to availability in the external dataset. Whether the same results can be obtained with the original 10 items requires further verification.

The survey asked the participants to subjectively answer questions about their health to measure health indicators. While it is a reasonable method for surveys with such a large sample size, some might be concerned that our indicators are subjective. Objective measurement of individual health would be more valid and reliable if examined with biomarker indicators (e.g., Kitayama et al., 2016). The health of employees is a concern for any organization, and the recent social trend of health management has increased the need to measure individual health objectively and with high accuracy. In addition, it is extremely important to consider specific approaches that contribute to the improvement of individual health by examining effective intervention methods for factors such as interpersonal relationships, job content, and personnel evaluation systems.

In recent years, government-led “work style reform” has promoted overtime regulations and the use of paid vacations, and the productivity and health of workers have been improving. However, it is also important to consider the “quality” of the work style in addition to the “quantity” approach. Here, we consider “quality” as the psychological motivation of individuals to work, such as job satisfaction. The authors believe that it is extremely important to create a work environment in which each worker can play an active role, as the working population in Japan is decreasing due to the declining birthrate and aging population.

## Data Availability Statement

The data analyzed in this study is subject to the following licenses/restrictions: Permission from Recruit Works Institute. Requests to access these datasets should be directed to https://www.works-i.com.

## Ethics Statement

Ethical review and approval was not required for the study on human participants in accordance with the local legislation and institutional requirements. The patients/participants provided their written informed consent to participate in this study.

## Author Contributions

SA and FK contributed equally on the research design, data collection, data analysis, and preparation of the manuscript. SY provided professional information and partially wrote the manuscript. All authors approved the submitted version.

## Conflict of Interest

The authors declare that the research was conducted in the absence of any commercial or financial relationships that could be construed as a potential conflict of interest.

## Publisher’s Note

All claims expressed in this article are solely those of the authors and do not necessarily represent those of their affiliated organizations, or those of the publisher, the editors and the reviewers. Any product that may be evaluated in this article, or claim that may be made by its manufacturer, is not guaranteed or endorsed by the publisher.
